# Combined Effects of High-Dose Bisphenol A and Oxidizing Agent (KBrO_3_) on Cellular Microenvironment, Gene Expression, and Chromatin Structure of Ku70-deficient Mouse Embryonic Fibroblasts

**DOI:** 10.1289/EHP237

**Published:** 2016-04-15

**Authors:** Natalie R. Gassman, Erdem Coskun, Pawel Jaruga, Miral Dizdaroglu, Samuel H. Wilson

**Affiliations:** 1 Genome Integrity and Structural Biology Laboratory, National Institute of Environmental Health Sciences, National Institutes of Health, Department of Health and Human Services, Research Triangle Park, North Carolina, USA; 2 Biomolecular Measurement Division, National Institute of Standards and Technology, Gaithersburg, Maryland, USA; 3 Faculty of Pharmacy, Gazi University, Ankara, Turkey

## Abstract

**Background:**

Exposure to bisphenol A (BPA) has been reported to alter global gene expression, induce epigenetic modifications, and interfere with complex regulatory networks of cells. In addition to these reprogramming events, we have demonstrated that BPA exposure generates reactive oxygen species and promotes cellular survival when co-exposed with the oxidizing agent potassium bromate (KBrO3).

**Objectives:**

We determined the cellular microenvironment changes induced by co-exposure of BPA and KBrO3 versus either agent alone.

**Methods:**

Ku70-deficient cells were exposed to 150 μM BPA, 20 mM KBrO3, or co-exposed to both agents. Four and 24 hr post-damage initiation by KBrO3, with BPA-only samples timed to coincide with these designated time points, we performed whole-genome microarray analysis and evaluated chromatin structure, DNA lesion load, glutathione content, and intracellular pH.

**Results:**

We found that 4 hr post-damage initiation, BPA exposure and co-exposure transiently condensed chromatin compared with untreated and KBrO3-only treated cells; the transcription of DNA repair proteins was also reduced. At this time point, BPA exposure and co-exposure also reduced the change in intracellular pH observed after treatment with KBrO3 alone. Twenty-four hours post-damage initiation, BPA-exposed cells showed less condensed chromatin than cells treated with KBrO3 alone; the intracellular pH of the co-exposed cells was significantly reduced compared with untreated and KBrO3-treated cells; and significant up-regulation of DNA repair proteins was observed after co-exposure.

**Conclusion:**

These results support the induction of an adaptive response by BPA co-exposure that alters the microcellular environment and modulates DNA repair. Further work is required to determine whether BPA induces similar DNA lesions in vivo at environmentally relevant doses; however, in the Ku70-deficient mouse embryonic fibroblasts, exposure to a high dose of BPA was associated with changes in the cellular microenvironment that may promote survival.

**Citation:**

Gassman NR, Coskun E, Jaruga P, Dizdaroglu M, Wilson SH. 2016. Combined effects of high-dose bisphenol A and oxidizing agent (KBrO3) on cellular microenvironment, gene expression, and chromatin structure of Ku70-deficient mouse embryonic fibroblasts. Environ Health Perspect 124:1241–1252; http://dx.doi.org/10.1289/EHP237

## Introduction

World-wide production of bisphenol A (BPA) has increased exponentially as the demand for this chemical in consumer products, from food and beverage containers to epoxies, has grown (Vandenberg et al. 2007). This increase has resulted in elevated BPA levels in the air, water, and soil, as well as in human samples (Vandenberg et al. 2007, 2010). The ubiquity of BPA in our environment has resulted in concurrent exposures of BPA with endogenous and exogenous DNA-damaging events. Together, these exposures can increase the damage load of genomic DNA and have implications for genomic stability and the development and progression of disease. The estrogenic properties of BPA are one source of concern, and BPA exposure has been shown to cause DNA damage independent of its estrogenic properties over a range of doses from environmentally relevant nanomolar to high micromolar concentrations in both *in vitro* and *in vivo* models (Iso et al. 2006; Nishimura et al. 2014; Tiwari et al. 2012; Wu et al. 2013; Yang et al. 2009). However, the ways in which the DNA damage response and repair pathways address BPA exposure have not been extensively investigated.

**Figure 1:**
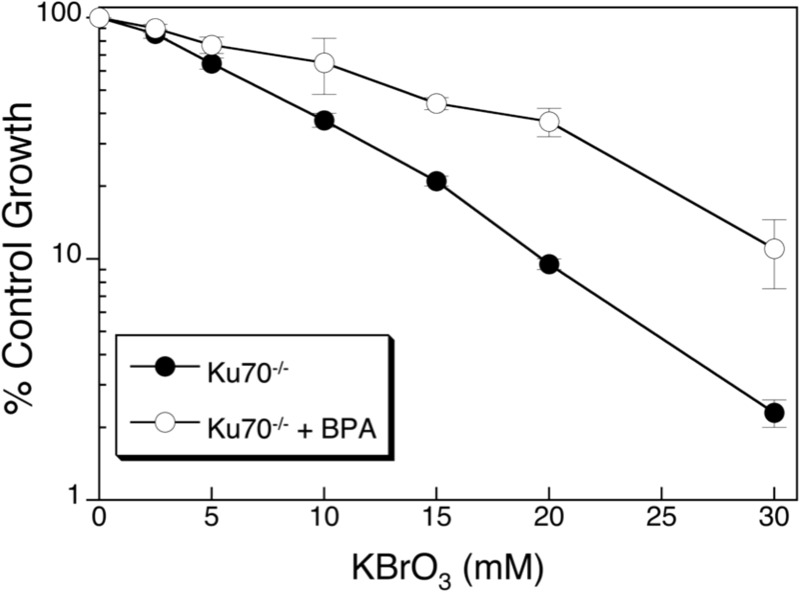
Cell survival following co-exposure to BPA and KBrO_3_ (from Gassman et al. 2015). Ku70-deficient cells were treated with increasing amounts of KBrO_3_ for 1 hr (solid circles) or pre-treated with 150 μM BPA for 1 hr, co-exposed to BPA and increasing amounts of KBrO_3_ for 1 hr, and then BPA exposure was continued for a total of 24 hr after KBrO_3_ exposure (open circles). After 24 hr, cells were washed, fresh medium was applied, and they were allowed to grow for 6–7 days. Cells were counted, and inhibition of growth was determined as the number of cells remaining after the treatment was compared with the control (% Control).

We previously demonstrated that exposure to high-dose BPA (150 μM) generates reactive oxygen species (ROS) in a model experimental system of Ku70-deficient mouse embryonic fibroblasts (MEFs) (Gassman et al. 2015). The Ku70-deficient cell line is sensitive to oxidizing agents, and its deficiency in double-strand break repair by nonhomologous end joining, which also serves as a back-up repair pathway for the base excision repair (BER) pathway, provides a window into the cellular responses to oxidatively induced DNA damage (Choi et al. 2014; Li et al. 2013). Using this repair-deficient cell line, high-dose BPA exposure was found to increase oxidatively induced DNA lesions in genomic DNA (Gassman et al. 2015). Because these BPA-induced DNA lesions would occur in concert with other DNA-damaging events during environmental exposures, the effects of co-exposure to BPA and the dietary oxidizing agent potassium bromate (KBrO_3_) were also examined. KBrO_3_, which is primarily used in flour and bread-making to improve elasticity and rising, induces ROS and oxidatively induced DNA lesions, and it has been shown to be a carcinogen at high doses in various animal models (Ballmaier and Epe 2006). Although KBrO_3_ has been banned in a number of countries, it continues to be used in the United States, and co-exposure with BPA from foodstuffs may occur. However, this co-exposure would occur at lower doses than described both here and in our previous work. We chose to examine the effects of a high dose of KBrO_3_ over a short exposure period to concentrate the effects of this agent. Using these conditions, we found that co-exposure to BPA and KBrO_3_ resulted in a further increase in the levels of oxidatively induced DNA lesions (Table 1). Both thymine glycol and 2,6-diamino-4-hydroxy-5-formamidopyrimidine (FapyGua) levels were significantly elevated over those of the control and over those of cells exposed to KBrO_3_ alone (Gassman et al. 2015). Surprisingly, despite the fact that both BPA and KBrO_3_ induce oxidative stress, an improvement in cellular survival was observed after co-exposure to both agents (Figure 1, from Gassman et al. 2015).

**Table 1:** Measured oxidatively damaged DNA bases in Ku70^–/–^ genomic DNA 4 hr post-damage induction*^a^* (adapted from Gassman et al. 2015).

Exposure	DNA lesion/10^6^ DNA bases (mean ± SD, *n* > 3)^*b*^			
	ThyGly	FapyAde	FapyGua	8-oxoGua
Control	3.82 ± 1.32	2.83 ± 0.98	3.69 ± 1.41	0.98 ± 0.16
BPA	8.41 ± 0.45*	3.72 ± 1.37	5.19 ± 0.92	1.21 ± 0.40
KBrO_3_	5.11 ± 0.69	4.01 ± 0.86	4.60 ± 0.92	1.33 ± 0.55
BPA + KBrO_3_	7.48 ± 0.48*^†^	4.38 ± 0.41*	6.77 ± 1.36*^†^	1.55 ± 0.59
^***a***^Samples treated with 150 μM BPA only were exposed for 5 hr; those treated with 20 mM KBrO_3_ only were exposed for 1 hr, then medium was replaced for 3 hr; and samples co-exposed to 150 μM BPA were exposed for 1 hr, then exposed to 150 μM BPA + 20 mM of KBrO_3_ for 1 hr, followed by exposure for 3 hr to 150 μM BPA only. ^***b***^Measurements were made by GC/MS as described in “Methods.” *p < 0.05 compared with untreated controls. ^†^*p* < 0.05 compared with KBrO_3_.				

Further examination of this cellular protective effect revealed that in the early repair window of 4 hr after exposure, BPA co-exposure reduced DNA strand-break signaling and resulted in the persistence of oxidatively induced DNA lesions (Table 1) (Gassman et al. 2015) beyond the typical DNA repair window of 2–4 hr (Hollenbach et al. 1999; Jaruga and Dizdaroglu 1996). This combination of reduced strand-break initiation coupled with increased lesion load is often observed in glycosylase-deficient cells, suggesting that BPA may prevent the initiation of repair of oxidized base lesions, thus reducing the production of toxic strand-break intermediates (Roth and Samson 2002; Sobol et al. 2003). Given that induction of oxidative stress can have profound consequences on the cellular microenvironment and that sustained oxidative insults have been shown to play a role in pathological processes, such as inflammation, cancer, and neurodegenerative diseases (Benhar et al. 2002; Roberts et al. 2009), understanding the mechanisms through which high-dose BPA co-exposure might influence cell survival and cell death *in vitro* may improve our understanding of the potential consequences of low-dose environmental exposure to BPA.

In the present study, we examined the cellular microenvironment 4 hr after BPA exposure, as in the study reported by Gassman et al. (2015), and extended the analysis to 24 hr after exposure. As in our previous study, a high dose of BPA over a short exposure period was used to concentrate the effects of BPA and to allow DNA damage and repair effects to be closely monitored. Owing to the efficiency and high catalytic responses of DNA repair proteins, a high-dose/short-duration exposure is often employed to observe DNA repair proteins in action, and our BPA and KBrO_3_ doses were selected with these parameters in mind. The high dose of BPA employed in this study was found to be minimally cytotoxic with no observed increase in cell proliferation (Gassman et al. 2015). Increases in cell proliferation after exposure to BPA are frequently observed at low doses (Lapensee et al. 2009; Pfeifer et al. 2015); however, our 150-μM dose of BPA did not increase proliferation in the MEF model system that we used (Gassman et al. 2015), consistent with other high-dose BPA studies (Ge et al. 2014; Samuelsen et al. 2001).

Whole-genome microarray analysis was performed to evaluate the global transcriptome changes associated with co-exposure to BPA and KBrO_3_ at these two time points and to identify gene targets promoting cell survival that are induced by co-exposure. Further, microenvironment changes in chromatin structure, glutathione content, and pH induced by exposure to BPA, KBrO_3_, or both agents were also examined at these time points to determine whether an adaptive response was induced by co-exposure.

## Methods

### Chemicals

BPA (Sigma Aldrich) was prepared in absolute ethanol and diluted to the final working concentration in medium. KBrO_3_ was dissolved directly in the medium at the time of the experiment.

### Cell Culture

Ku70^–/–^ mouse embryonic fibroblasts (MEFs) (a gift from Dr. Shigemi Matsuyama, Case Western University, Cleveland, OH) were grown at 37°C in a 10% CO_2_ incubator in Dulbecco’s modified Eagle’s medium (DMEM) supplemented with glutamine, 10% fetal bovine serum (FBS; HyClone), 1% nonessential amino acids, and 1% sodium pyruvate (Gama et al. 2009). Cells were routinely tested and were found to be free of mycoplasma contamination.

### Cytotoxicity Studies

Cytotoxicity was determined by growth inhibition assays. We consider this cell survival assay to be more reliable in MEFs than alternate assays such as clonogenic colony counting or short-term cell killing assays. Results obtained with the cell survival assay were confirmed using other assays. Cells were seeded in six-well dishes at a density of 40,000 cells/well. The following day, cells were exposed to a range of concentrations of BPA alone for 25 hr or to KBrO_3_ alone for 1 hr. In other cases, cells were exposed to 150 μM BPA for 1 hr, then a range of KBrO_3_ concentrations for 1 hr, and finally with 150 μM BPA for a further 23 hr. For KBrO_3_ alone and for BPA plus KBrO_3_ co-exposures, after the 1 hr KBrO_3_ treatment, the cells were washed with Hanks’ balanced salt solution (HBSS), and fresh medium was added with or without BPA. After a 25-hr exposure to BPA, cells were washed with HBSS, and fresh medium was added. Dishes were then incubated for 6–7 days at 37°C in a 10% CO_2_ incubator until the untreated control cells were approximately 80% confluent. Cells (triplicate wells for each drug concentration) were counted by a cell lysis procedure (Butler 1984), and the results were expressed as the number of cells in drug-treated wells relative to the number of cells in control wells (% control growth).

### RNA Isolation

Ku70^–/–^ cells were seeded in 145-mm dishes at 1 × 10^6^ cells/dish and were cultured to 80% confluence. Cells were then treated with BPA or KBrO_3_ or were co-exposed to BPA and KBrO_3_. KBrO_3_-only cells were treated for 1 hr with 20 mM KBrO_3_, washed with HBSS, and then, fresh medium was added to the cells. Cells were allowed to repair for an additional 3 or 23 hr following treatment. For the BPA-only treatment, cells were incubated for 5 or 25 hr in medium containing 150 μM BPA. For co-exposure, cells were first incubated with 150 μM BPA for 1 hr and then with 20 mM KBrO_3_ and 150 μM BPA for 1 hr; then, the cells were washed with HBSS, fresh medium with 150 μM BPA was added, and the cells were allowed to repair for an additional 3 or 23 hr. Because all treatments were conducted in parallel, the post-damage induction times throughout the manuscript refer to the initiation of KBrO_3_ treatment, although no KBrO_3_ was added to the BPA-only cells. Four and 24 hr after KBrO_3_ treatment, cells were washed twice in phosphate-buffered saline (PBS; Hyclone), and total cellular RNA was isolated using an RNeasy Midi Kit (Qiagen) according to the manufacturer’s instructions. Residual genomic DNA was removed by on-column digestion with RNase-free DNase I (Qiagen). Denaturing formaldehyde/agarose gel electrophoresis was performed to validate the quality and integrity of the RNA samples, the samples were quantified using a Nanodrop ND-1000 spectrophotometer (Thermo Scientific), and sample purity was assessed by calculating the 260:280 absorbance ratio. Three biological replicates were collected and isolated for the control and for all of the treatment conditions.

### Microarray Study

Gene expression analysis was performed using Agilent Whole Mouse Genome 4 × 44 multiplex format oligo arrays (Agilent Technologies) following the Agilent one-color microarray-based gene expression analysis protocol. Starting with 500 ng of total RNA, Cy3-labeled cRNA was produced according to the manufacturer’s protocol. For each sample, 1.65 μg of Cy3-labeled cRNA was fragmented and hybridized for 17 hr in a rotating hybridization oven. Slides were washed and then scanned with an Agilent Scanner. Data were obtained with Agilent Feature Extraction software (v9.5), using the one-color defaults for all parameters. The Agilent Feature Extraction Software performed error modeling, adjusting for additive and multiplicative noise. The resulting data were processed using Omicsoft Array Studio software (v.7.0). Significant probes were determined by filtering data to include only probes with fold changes > 1.5 or < –1.5 compared with the control and *p-*values < 0.01, which was determined by an error-weighted one-way analysis of variance (ANOVA) and Bonferroni multiple test correction using the Omicsoft software. This list of differentially expressed genes generated by the Omicsoft software was used as an input for the curated pathway database, Ingenuity Pathway Analysis (IPA; Ingenuity® Systems; http://www.ingenuity.com). IPA’s Core Analysis module used the differentially expressed gene list to enrich for canonical and functional pathways or regulatory connections and to remove duplicates and unmapped genes. Significance values were calculated using a right-tailed Fisher’s exact test to determine if a pathway was overrepresented by calculating whether genes in a specific pathway were enriched within the data set compared with all genes on the array in the same pathway at a *p* < 0.05 cutoff for significance based on IPA threshold recommendations. Only pathways with a *p-*value exceeding the threshold and with more than two representative genes in the data set were considered. Final filtered gene lists generated by IPA were input into Partek® Genomic Suite software to create heat maps of hierarchical clustered genes and into http://www.pangloss.com/seidel/Protocols/venn.cgi to create Venn diagrams. Microarray intensity files can be accessed through Gene Expression Omnibus (http://www.ncbi.nlm.nih.gov/geo/) with the accession number GSE71489.

### Chromatin Condensation

The level of chromatin condensation was measured using the Hoechst, YO-PRO1, and propidium iodide (PI) stains from the Chromatin Condensation & Membrane Permeability Dead Cell Apoptosis Kit (Life Technologies), similarly to the method reported by Muders et al. (2009). The intensity of Hoechst staining of DNA in compact chromatin regions, such as heterochromatin, is much more intense then Hoechst intercalated into looser regions of chromatin, such as euchromatin; therefore, the difference in Hoechst intensity can be used to determine the degree of compaction observed across nuclei (Belloc et al. 1994; Hinde et al. 2014; Mora-Bermúdez and Ellenberg 2007; Zink et al. 2003). Examination of Hoechst intensity across nuclei with confocal microscopy has been used to observe chromatin dynamics in living cells, and here, we extend this type of intensity measure with flow cytometry with the addition of YO-PRO1 and PI staining to identify apoptotic or necrotic cells during analysis. Ku70^–/–^ cells were seeded in 100-mm dishes at a density of 1 × 10^6^ cells/dish and then were treated on the following day with BPA, KBrO_3_, or co-exposed, as described above. Four or 24 hr after the initiation of KBrO_3_ treatment, cells were harvested using 0.25% trypsin, washed in 5 mL of PBS, and stained in 1 mL of PBS with 1 μL each of Hoechst 33342 stock solution, YO-PRO-1 stock solution, and PI stock solution at 23°C for 15 min. Staurosporine-treated cells were analyzed as a control for condensed chromatin. Cells were incubated with 2 μM staurosporine for 4 hr at 37°C and then were harvested and stained as described for BPA, KBrO_3_, or co-exposed samples. Stained cells were analyzed with a Becton Dickinson LSRII flow cytometer (BD). The variations in Hoechst staining intensity in the live cells were plotted. The mean fluorescence intensity was reported for the Hoechst channel, which was the only channel to show staining and measurable changes in mean fluorescence intensity for BPA, KBrO3, and co-exposure conditions. Staurosporine-treated cells showed staining in all three channels. The mean intensities ± standard error of the mean (SEM) for at least three experiments are reported.

### Reduced Glutathione Assay

Levels of cellular reduced glutathione (GSH) were analyzed using ThiolTracker™ Violet GSH detection reagent (Life Technologies) according to the manufacturer’s protocol. Ku70^–/–^ cells were seeded in 100-mm dishes at a density of 1 × 10^6^ cells/dish and were treated as described above. Four or 24 hr after the initiation of KBrO_3_ treatment, cells were harvested using 0.25% trypsin, washed in 4 mL of PBS, and stained in PBS containing 10 μM ThiolTracker™ Violet for 30 min at 37°C. Stained cells were analyzed by flow cytometry on an LSRII flow cytometer, and the mean fluorescence intensity was recorded for ThiolTracker™ Violet. The mean intensities ± SEM of three experiments are reported.

### pH Measurement

Intracellular pH was quantified by flow cytometry with the pHRodo® Red AM Intracellular pH Indicator (Life Technologies) according to the manufacturer’s protocol. pHRodo® is weakly fluorescent at neutral pH and is increasingly fluorescent in acidic pH with a detection range between 4 and 9. Ku70^–/–^ cells were seeded in 100-mm dishes at a density of 1 × 10^6^ cells/dish and were then treated as described above with BPA or KBrO_3_ or were co-exposed to both agents. Additionally, for every experiment, a calibration curve was prepared using an Intracellular pH Calibration Buffer Kit (Life Technologies). Four or 24 hr after the initiation of KBrO_3_ treatment, cells were harvested using 0.25% trypsin, washed in 4 mL of PBS, and stained with pHRodo® Red at 37°C for 30 min. Cells were then washed twice in PBS, and the calibration curve samples were resuspended in valinomycin and nigericin with pH calibration buffers of pH 5.5, 6.5, and 7.5 for 5 min before analysis, according to the manufacturer’s protocol. The addition of valinomycin and nigericin assists in the equilibration of the intracellular space with the pH buffer. Samples were then analyzed by flow cytometry on an LSRII flow cytometer, and the mean fluorescence intensity was recorded for pHRodo® Red. A standard curve was prepared using the calibration buffer intensities, and the pH values were calculated for the control and treated samples. The mean pH values ± SEM calculated for four experiments are reported.

### Measurement of Oxidatively Induced DNA Lesions

Gas chromatography/tandem mass spectrometry (GC-MS/MS) with isotope dilution was used to identify and quantify modified DNA bases in DNA as described previously (Gassman et al. 2015).

### Statistical Analysis

Measured DNA lesions are expressed as the mean ± standard deviation (SD), and all other values are expressed as the mean ± standard error of the mean (SEM). The data were analyzed by ANOVA and Tukey post hoc analysis. *p-*Values < 0.05, denoted by * or †, were considered to correspond with statistical significance.

## Results

### Whole Genome Microarray and Pathway Analysis

To examine cellular changes induced by BPA, KBrO_3_, and the co-exposure conditions, we performed whole genome microarray analysis of cells treated with BPA, KBrO_3_, or co-exposed to BPA and KBrO_3_. KBrO_3_-only cells were treated for 1 hr with 20 mM KBrO_3_, washed, and allowed to repair for an additional 3 or 23 hr following treatment. For BPA-only treatment, cells were incubated for 5 or 25 hr in medium containing 150 μM BPA. For co-exposure, cells were incubated with 150 μM BPA for 1 hr and then with 20 mM KBrO_3_ and 150 μM BPA for 1 hr, after which the cells were washed with HBSS; then, fresh medium with 150 μM BPA was added, and cells were allowed to repair for an additional 3 or 23 hr. Treatments were performed in parallel, and repair times after exposures are expressed relative to the initiation of KBrO_3_ treatment, even though no KBrO_3_ was added in the BPA-only treatment.

**Figure 2:**
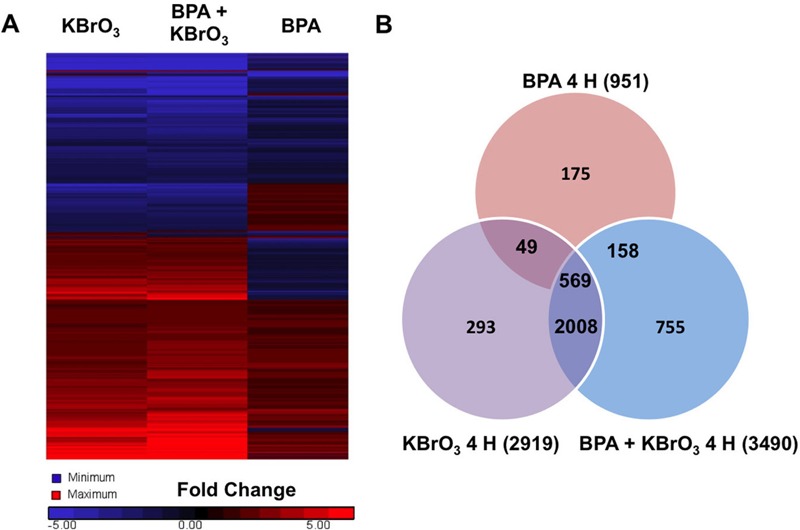
Gene expression changes observed by whole genome analysis of mRNA isolated 4 hr after treatment with KBrO_3_, BPA, or co-exposure to both agents as described in “Methods.” (*A*) Heat map of gene expression changes observed after treatment was generated using Partek^®^ Genomic Suite software with probes selected by a fold change cutoff of ± 1.5 compared with untreated controls and an analysis of variance (ANOVA)-calculated significance level of *p* < 0.01 (*n* = 3). (*B*) Significant probe changes identified using the described criteria are sorted by Venn diagram.

**Figure 3:**
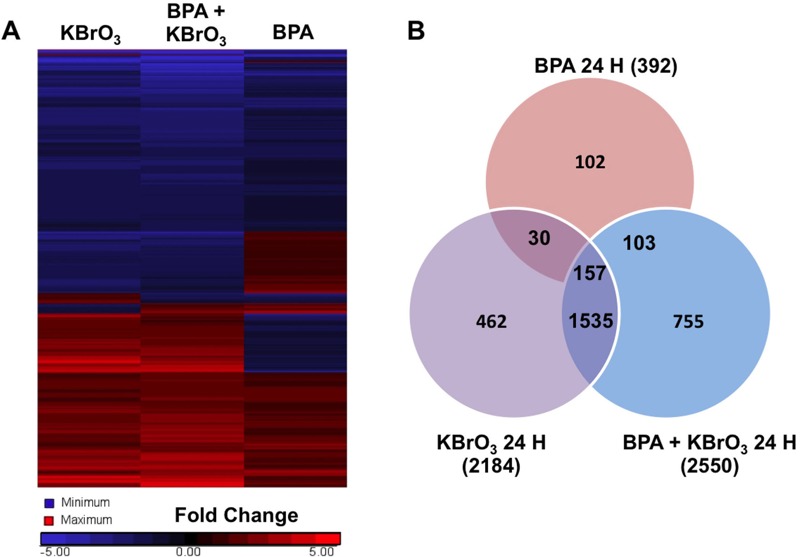
Gene expression changes observed by whole genome analysis of mRNA isolated 24 hr after treatment with KBrO_3_, BPA, or co-exposure to both agents, as described in “Methods.” (*A*) Heat map of gene expression changes observed after treatment was generated using Partek^®^ Genomic Suite software with probes selected by a fold change cutoff of ± 1.5 compared with untreated controls and an analysis of variance (ANOVA)-calculated significance level of *p* < 0.01 (*n* = 3). (*B*) Significant probe changes identified using the described criteria are sorted by Venn diagram.

Gene lists were generated from the average of three biological replicates for each condition, and significant probes were identified by selecting those probes showing a *p* value < 0.01, as determined by error-weighted ANOVA with Bonferroni multiple-test correction. Duplicate reads and noncoding genes were removed by IPA software. At 4 hr post-damage induction, 4,007 unique genes were altered after treatment with KBrO_3_, BPA, or after co-exposure, and 3,144 unique gene changes were observed 24 hr after damage induction. Figure 2A shows a heat map of the observed gene expression changes 4 hr after treatment, and Figure 3A shows the observed changes at 24 hr. Figure 2B shows a Venn diagram analysis of the gene list 4 hr post-damage induction and illustrates the common and unique gene expression changes among the treatment groups. Figure 3B shows the Venn diagram of these changes at 24 hr post-damage induction.

At 4 hr post-damage induction, 2,008 genes were significantly different from controls after treatment with KBrO_3_ only and with BPA + KBrO_3_, and 569 genes were significantly different from controls after all three treatments (Figure 2). IPA was used to identify the top five networks that were significantly regulated in response to each treatment (Appendix 1, with networks ranked based on score and number of focus molecules), and the top five up-regulated and top five down-regulated genes for each treatment are shown in Table 2 (with rankings based on fold change over control with a *p*-value < 0.01). Appendix 2 and Table 3 show the top-ranked networks and induced and repressed genes observed after 24 hr. There are no common top networks associated with each treatment at 4 or 24 hr; however, there are common top genes induced or repressed at each time point. Cells treated with KBrO_3_ alone and co-exposed to BPA both showed up-regulation of *GSTA5* at both 4 and 24 hr; up-regulation of *IL18R* was observed at 4 hr, and up-regulation of *ROBO3* was observed at 24 hr. Down-regulation of *Cyp2d22* and *Akr1b10* at 4 hr and of *HP* and *CYP2F1* at 24 hr was also observed for both KBrO_3_-containing treatments. Up-regulation of *EGR4* at 4 hr was the only common change observed for BPA-only and KBrO_3_-only treatments. Overall, each treatment condition altered network signaling and gene expression in a different manner, with the greatest overlap observed for the KBrO_3_ and co-exposure conditions, as illustrated by the heat maps and Venn diagrams (Figures 2 and 3).

**Table 2:** Top five up-regulated and top five down-regulated genes ranked by magnitude of fold change over control at 4 hr post-damage induction.*^a^*

Outcome	BPA		KBrO_3_		BPA + KBrO_3_	
	Gene	Fold change^*b*^	Gene	Fold change	Gene	Fold change
Up-regulated	*CCL20*	60.370	*GSTA5*	195.423	*IL18R1*	150.272
	*CXCL3*	55.581	*IL18R1*	165.986	*GSTA5*	118.712
	*Saa3*	26.361	*AREG*	131.227	*PTGS2*	95.172
	*HCAR2*	25.405	*DUSP2*	92.477	*AREG*	84.638
	*EGR4*	23.721	*EGR4*	65.351	*ATF3*	81.490
Down-regulated	*LGALS12*	–10.284	*KLF15*	–56.007	*TNS1*	–41.975
	*DSC1*	–8.749	*Cyp2d22*	–35.115	*Akr1b10*	–39.628
	*FRY*	–5.689	*IKZF2*	–22.961	*FZD2*	–35.905
	*HPGD*	–4.937	*BMF*	–22.292	*Cyp2d22*	–35.268
	*PLCH2*	–4.768	*Akr1b10*	–21.197	*KAT2B*	–30.719
^***a***^Samples treated with 150 μM BPA only were exposed for 5 hr; those treated with 20 mM KBrO_3_ only were exposed for 1 hr, then medium was replaced for 3 hr; and samples co-exposed to 150 μM BPA were exposed for 1 hr, then exposed to 150 μM BPA + 20 mM of KBrO_3_ for 1 hr, followed by exposure for 3 hr to 150 μM BPA only. ^***b***^Gene expression fold change was evaluated in comparison with untreated control with all *p*-values < 0.01.						

**Table 3:** Top five up-regulated and top five down-regulated genes ranked by magnitude of fold change over control at 24 hr post-damage induction.*^a^*

Outcome	BPA		KBrO_3_		BPA + KBrO_3_	
	Gene	Fold change^*b*^	Gene	Fold change	Gene	Fold change
Up-regulated	*Wfdc17*	37.229	*GSTA5*	87.219	*GSTA5*	181.836
	*Saa3*	25.573	*ROBO3*	22.114	*Prg4*	62.482
	*LCN2*	19.522	*Prg4*	16.538	*ROBO3*	59.949
	*OSTN*	16.420	*BLNK*	15.800	*MMP15*	25.597
	*CA6*	15.788	*PTPN22*	15.395	*CA6*	23.355
Down-regulated	*MYH1*	–11.145	*FGL2*	–73.599	*AGTR2*	–153.275
	*Nebl*	–10.875	*CYP2F1*	–59.905	*HP*	–125.757
	*MYH2*	–9.608	*SLCO2B1*	–57.705	*DIO3*	–114.982
	*SLC26A7*	–9.425	*VIT*	–55.675	*SLCO2B1*	–102.011
	*NPR3*	–9.109	*HP*	–37.549	*CYP2F1*	–85.326
^***a***^Samples treated with 150 μM BPA only were exposed for 25 hr; those treated with 20 mM KBrO_3_ only were exposed for 1 hr, then medium was replaced for 23 hr; and co-exposed samples were exposed to 150 μM BPA for 1 hr, then exposed to 150 μM BPA + 20 mM of KBrO_3_ for 1 hr, followed by exposure for 23 hr to 150 μM BPA only. ^***b***^Gene expression fold change was evaluated in comparison with untreated control with all *p*-values < 0.01.						

A total of 755 genes were significantly different from controls in co-exposed cells at 4 hr, and 755 genes were also significantly different at 24 hr, but only 86 genes were common to both time points. IPA was performed on the 669 significant genes that were unique to co-exposed cells at 4 hr, and on the 669 significant genes that were unique to co-exposed cells at 24 hr, and the top five networks based on the unique genes at each time point are shown in Appendix 3.

**Figure 4:**
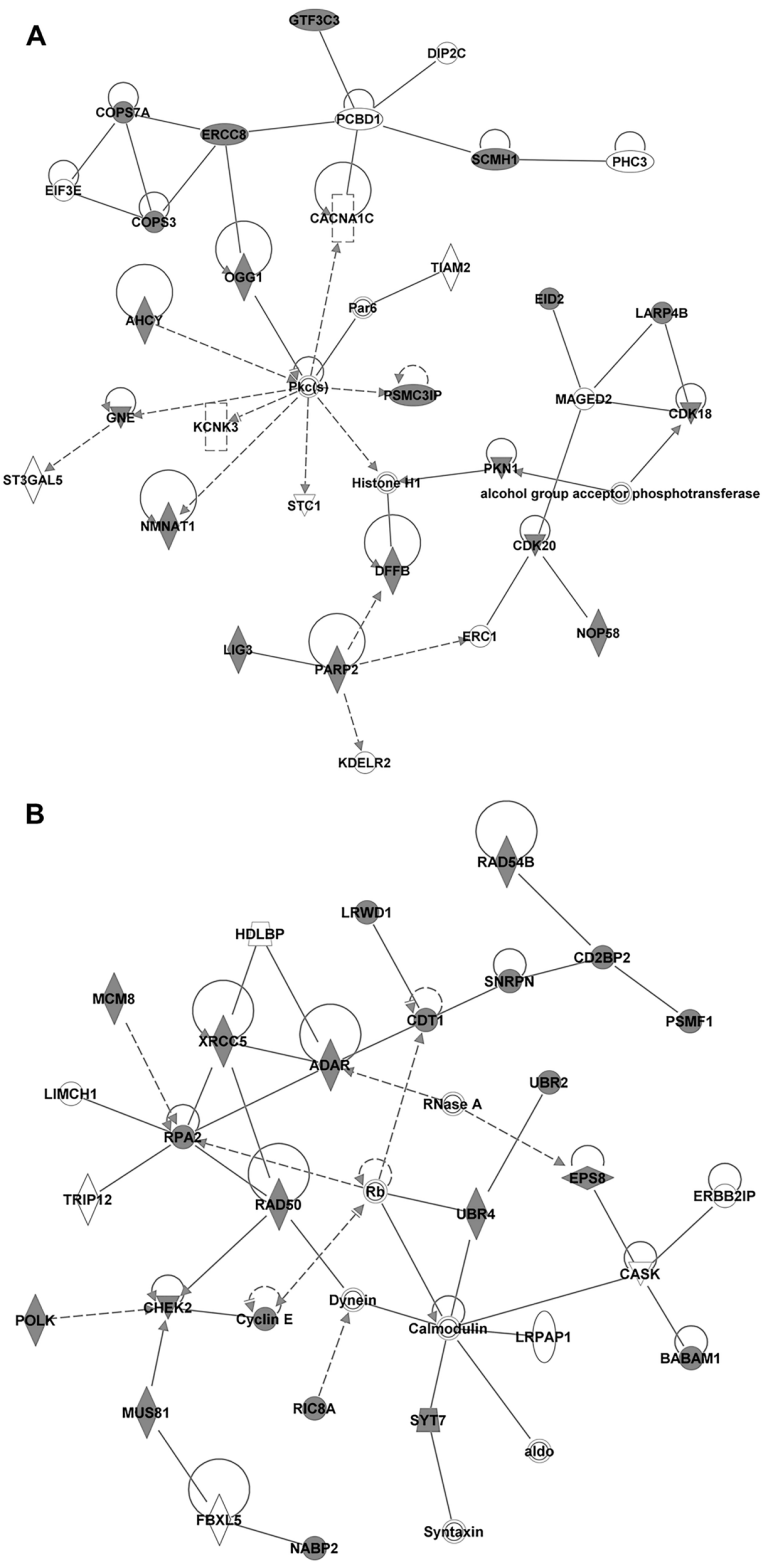
DNA replication, recombination, and repair networks identified from the uniquely regulated genes identified from the co-exposure condition 24 hr after damage induction by IPA’s Core Analysis module, which searches for enriched canonical pathways. (*A*) DNA replication, recombination, and repair network 1 (score 46, 31 focus molecules, *p*-value of top functions 7.18 × 10^–5^) is presented with expression values for the co-exposure overlaid, as an indicator of up- or down-regulation (dark gray and white, respectively). (*B*) DNA replication, recombination, and repair network 3 (score 38, 28 focus molecules, *p* value of top functions 4.458 × 10^–8^) is presented with expression values for the co-exposure overlaid, as an indicator of up- or down-regulation (dark gray and white, respectively).

Two of the top five networks identified by IPA that were unique to the co-exposure condition at 24 hr are DNA Replication, Recombination networks (Appendix 3), and Figure 4A and B show these networks. Genes regulated by co-exposure in these networks include members of the base excision repair (BER), the nucleotide excision repair (NER), and double-strand break repair pathways. To better understand the gene expression changes induced by the three treatment conditions after 24 hr, we examined common DNA repair genes involved in the repair of oxidatively induced DNA damage from the BER, NER, and double-strand break repair pathways (Table 4). After 24 hr of treatment with BPA alone, no significant gene expression changes over control were observed in these genes, with *Rad51* being the only exception. Cells treated with KBrO_3_ alone and co-exposed cells showed differential expression of these repair genes, with significant changes observed after co-exposure for the BER proteins *Apex1*, *Lig3*, *Pnkp*, *Ogg1*, and *Tdp1*, and for the NER proteins *ERCC4*, *ERCC5*, and *ERCC8*. Table 5 shows the expression of these genes 4 hr after the induction of DNA damage with KBrO_3_. As shown in Table 5, treatment with BPA alone again had little effect on the expression of DNA repair genes, whereas differential expression was observed for cells treated with KBrO_3_ alone and those co-exposed to BPA. Interestingly, there was suppression of a number of genes involved in the removal of oxidatively induced DNA lesions, such as *Ogg1*, *Neil1*, and *Neil3*, and of genes involved in the subsequent gap-filling reaction, *PolB* and *PolL*. With the exception of *Ogg1*, for which changes in gene expression were observed at both 4 and 24 hr, these changes were unique to the 4-hr time point. These observed changes in gene expression at both 4 and 24 hr are consistent with BPA co-exposure inducing an adaptive response through gene regulation changes after the 4-hr time point.

**Table 4:** Selected DNA repair genes identified at 24 hr post-damage induction*^a^*, with the magnitude of the fold change over control for genes with *p* values < 0.05.

BPA	Fold change^*b*^	KBrO_3_	Fold change	BPA + KBrO_3_	Fold change
*Apex1*	n.c.	*Apex1*	1.67	*Apex1*	1.82
*Ercc3*	n.c.	*Ercc3*	2.09	*Ercc3*	1.86
*Ercc5*	n.c.	*Ercc5*	n.c.	*Ercc5*	1.85
*Ercc6*	n.c.	*Ercc6*	1.91	*Ercc6*	n.c.
*Ercc8*	n.c.	*Ercc8*	2.30	*Ercc8*	2.50
*Exo1*	n.c.	*Exo1*	2.14	*Exo1*	2.19
*Fen1*	n.c.	*Fen1*	2.64	*Fen1*	2.40
*Lig3*	n.c.	*Lig3*	n.c.	*Lig3*	2.02
*Lig4*	n.c.	*Lig4*	1.88	*Lig4*	n.c.
*Mre11*	n.c.	*Mre11*	2.89	*Mre11*	2.27
*Mutyh*	n.c.	*Mutyh*	2.22	*Mutyh*	1.64
*Ogg1*	n.c.	*Ogg1*	n.c.	*Ogg1*	1.86
*Parp1*	n.c.	*Parp1*	1.93	*Parp1*	1.68
*Rad51*	1.83	*Rad51*	2.13	*Rad51*	2.35
*Rpa1*	n.c.	*Rpa1*	1.47	*Rpa1*	2.35
*Tdg1*	n.c.	*Tdg1*	2.15	*Tdg1*	2.10
*Tdp1*	n.c.	*Tdp1*	1.44	*Tdp1*	1.66
Genes unique to 24 hr
*Atm*	n.c.	*Atm*	1.64	*Atm*	1.89
*Brca1*	n.c.	*Brca1*	2.62	*Brca1*	2.00
*Ercc1*	n.c.	*Ercc1*	2.07	*Ercc1*	1.70
*Ercc4*	n.c.	*Ercc4*	3.15	*Ercc4*	4.05
*Pnkp*	n.c.	*Pnkp*	n.c.	*Pnkp*	2.05
*PolD*	n.c.	*PolD*	n.c.	*PolD*	1.68
*PolK*	n.c.	*PolK*	n.c.	*PolK*	1.68
*Rad50*	n.c.	*Rad50*	1.58	*Rad50*	1.69
*Xrcc5*	n.c.	*Xrcc5*	n.c.	*Xrcc5*	1.92
n.c., No significant change over control. ^***a***^Samples treated with 150 μM BPA only were exposed for 25 hr; those treated with 20 mM KBrO_3_ only were exposed for 1 hr, then medium was replaced for 23 hr; and samples co-exposed to 150 μM BPA were exposed for 1 hr, then exposed to 150 μM BPA + 20 mM of KBrO_3_ for 1 hr, followed by exposure for 23 hr to 150 μM BPA only. ^***b***^Gene expression fold change was evaluated in comparison with untreated control with all *p*-values < 0.05.					

**Table 5:** Selected DNA repair genes identified 4 hr post-damage induction*^a^*, with the magnitude of the fold change over control for genes with *p* values < 0.05.

BPA	Fold change^*b*^	KBrO_3_	Fold change	BPA + KBrO_3_	Fold change
*Apex1*	n.c.	*Apex1*	n.c.	*Apex1*	1.58
*Ercc3*	n.c.	*Ercc3*	n.c.	*Ercc3*	1.45
*Ercc5*	n.c.	*Ercc5*	–1.49	*Ercc5*	n.c.
*Ercc6*	n.c.	*Ercc6*	n.c.	*Ercc6*	2.62
*Ercc8*	n.c.	*Ercc8*	4.63	*Ercc8*	6.36
*Exo1*	1.60	*Exo1*	2.33	*Exo1*	3.40
*Fen1*	n.c.	*Fen1*	1.73	*Fen1*	1.90
*Lig3*	n.c.	*Lig3*	n.c.	*Lig3*	2.02
*Lig4*	n.c.	*Lig4*	n.c.	*Lig4*	2.4
*Mre11*	n.c.	*Mre11*	n.c.	*Mre11*	2.27
*Mutyh*	n.c.	*Mutyh*	–3.33	*Mutyh*	–2.20
*Ogg1*	n.c.	*Ogg1*	–1.68	*Ogg1*	–2.00
*Parp1*	n.c.	*Parp1*	–1.28	*Parp1*	–1.39
*Rad51*	1.83	*Rad51*	–3.11	*Rad51*	–2.49
*Rpa1*	n.c.	*Rpa1*	1.56	*Rpa1*	1.53
*Tdg1*	n.c.	*Tdg1*	2.12	*Tdg1*	2.82
*Tdp1*	n.c.	*Tdp1*	1.44	*Tdp1*	1.66
Genes unique to 4 hr
*Mpg*	n.c.	*Mpg*	–1.91	*Mpg*	–2.59
*Neil1*	n.c.	*Neil1*	–2.48	*Neil1*	–2.46
*Neil3*	n.c.	*Neil3*	–6.24	*Neil3*	–11.36
*PolB*	n.c.	*PolB*	n.c.	*PolB*	–2.90
*PolL*	n.c.	*PolL*	–3.49	*PolL*	–2.92
*Xrcc1*	n.c.	*Xrcc1*	–1.72	*Xrcc1*	–1.76
n.c., No significant change over control. ^***a***^Samples treated with 150 μM BPA only were exposed for 5 hr; those treated with 20 mM KBrO_3_ only were exposed for 1 hr, then medium was replaced for 3 hr; and samples co-exposed to 150 μM BPA were exposed for 1 hr, then exposed to 150 μM BPA + 20 mM of KBrO_3_ for 1 hr, followed by exposure for 3 hr to 150 μM BPA only. ^***b***^Gene expression fold change was evaluated in comparison with untreated control with all *p*-values < 0.05.					

### Chromatin Condensation

Given the observed reduction in gene expression associated with BER and NER at 4 hr and our previous results indicating an increase in oxidatively induced DNA lesions coupled with a reduction in DNA strand breaks (Table 1) (Gassman et al. 2015), DNA repair may be altered during the first 4 hr of exposure, when repair of oxidatively induced DNA lesions typically occurs (Hollenbach et al. 1999; Jaruga and Dizdaroglu 1996). Chromatin structure has been demonstrated to regulate the access of DNA repair proteins to sites of DNA damage, and alterations in the chromatin structure have been shown to reduce the excision of lesions, such as 8-oxoGua (Amouroux et al. 2010).

**Figure 5:**
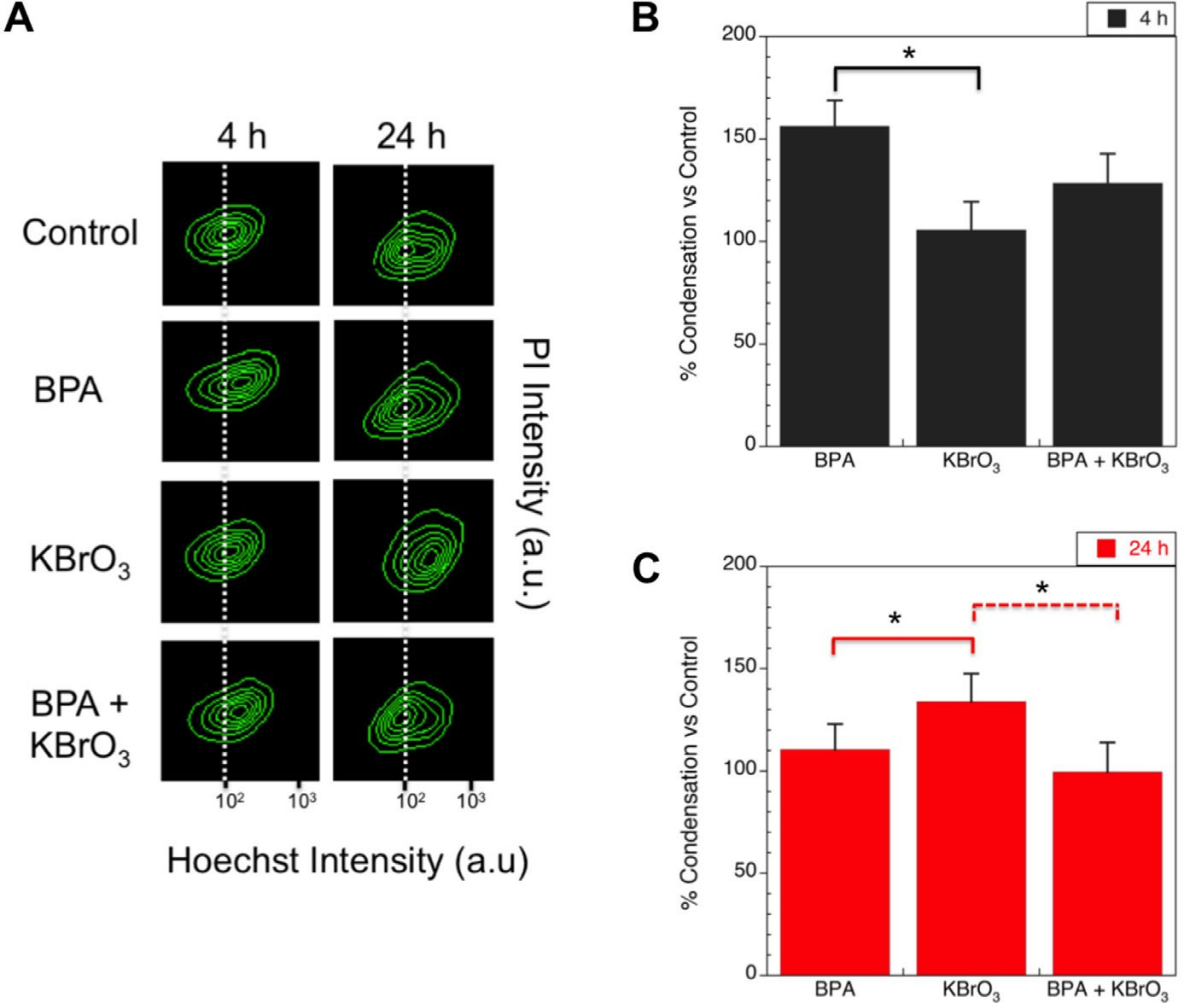
Levels of chromatin condensation after treatment with KBrO_3_, BPA, or co-exposure of both agents at 4 and 24 hr post-damage induction were measured by intensity of Hoechst and PI staining using flow cytometry. (*A*) Hoechst- and propidium iodide (PI)-stained live cells are sorted by intensity, and the contour maps of the measured intensities for a representative experiment at 4 and 24 hr are shown. Dashed lines show the center of the control contour plot and highlight changes relative to the control cells. (*B*) Mean intensity values of the Hoechst staining for each treatment condition 4 hr post-damage induction normalized to the control are shown (mean ± SEM of 3 biological replicates). (*C*) Mean intensities of the Hoechst staining for each treatment condition 24 hr post-damage induction normalized to the control are shown (mean ± SEM). *p < 0.05, with solid and dashed lines showing comparison groups.

To evaluate chromatin structure after BPA exposure and after co-exposure, we used a DNA-intercalating dye, Hoechst 33342, and flow cytometry to examine the degree of compaction. Hoechst 33342 brightly stains condensed chromatin (e.g., heterochromatin)and dimly stains looser transcription-ready chromatin (e.g., euchromatin) in live cells (Belloc et al. 1994; Khurana et al. 2014; Mora-Bermúdez and Ellenberg 2007; Zink et al. 2003). In this assay, the intensity of Hoechst staining in treated cells that are not undergoing apoptosis is measured, as indicated by the lack of YO-PRO and PI staining, and any observed shift in the mean intensity of the Hoechst dye then reveals the degree of chromatin compaction induced by treatment (Figure 5). With this technique, chromatin compaction was observed 4 hr after treatment with BPA alone and after co-exposure to BPA and KBrO_3_ (156% ± 12.5% and 128% ± 14.4% of control, respectively). This compaction may prevent DNA glycosylases from accessing oxidatively induced DNA lesions and is consistent with the lesion persistence and strand-break signaling reduction that have previously been reported (Gassman et al. 2015).

At 24 hr, the degree of chromatin compaction in BPA-treated cells was reduced significantly from that at the 4-hr time point, but it remained slightly higher than in untreated cells (110% ± 2.7% of control). In contrast, the KBrO_3_-treated cells showed compaction (133 ± 5.3% of control) at 24 hr, indicating that KBrO_3_-treated cells may have started to undergo apoptosis. Finally, co-exposed cells were consistent with untreated cells (99.6 ± 6.5%).

### Oxidatively Induced DNA Damage

Previously, we determined that a significant amount of oxidatively induced DNA lesions persisted in genomic DNA 4 hr after DNA damage induction [Table 1 and (Gassman et al. 2015)], and this retention of lesions is consistent with the reduced access of DNA glycosylases to DNA lesions in condensed chromatin (Amouroux et al. 2010). Because this compaction was reduced 24 hr after co-exposure, and because the microarray analysis supported the up-regulation of DNA repair genes, we quantified oxidatively induced DNA lesions in DNA isolated from treated cells 24 hr after damage induction to determine whether an increase in DNA lesions could still be observed 24 hr after treatment. GC-MS/MS with isotope dilution, as described by Gassman et al. (2015) and by Reddy et al. (2013), was used to quantify lesions in isolated nuclear DNA. The mean ± SD values for the quantified DNA lesions are summarized in Table 6.

**Table 6:** Levels of oxidatively induced DNA bases in Ku70^–/–^ genomic DNA 24 hr after damage induction*^a^.*

Exposure	DNA lesion/10^6^ DNA bases (mean ± SD, *n* > 3)^*b*^			
	ThyGly	FapyAde	FapyGua	8-oxoGua
Control	5.32 ± 0.80	3.61 ± 0.26	3.81 ± 0.83	3.25 ± 0.01
BPA	2.54 ± 0.69	2.75 ± 1.74	3.71 ± 1.88	3.09 ± 0.57
KBrO_3_	3.54 ± 0.27	3.21± 0.32	3.77 ± 1.31	2.84 ± 0.55
BPA + KBrO_3_	5.38 ± 1.74	3.35 ± 0.31	6.00 ± 2.02	2.80 ± 0.80
^***a***^Samples treated with 150 μM BPA only were exposed for 25 hr; those treated with 20 mM KBrO_3_ only were exposed for 1 hr, then medium was replaced for 23 hr; and co-exposed samples were exposed to 150 μM BPA for 1 hr, then exposed to 150 μM BPA + 20 mM of KBrO_3_ for 1 hr, followed by exposure for 23 hr to 150 μM BPA only. ^***b***^Measurements were made by GC/MS as described in “Methods.”				

Our previous results showed a significant accumulation of lesions in cells treated with BPA alone (ThyGly) and in cels co-exposed to BPA and KBrO_3_ (ThyGly, FapyAde, and FapyGua) 4 hr after damage induction [Table 1 and (Gassman et al. 2015)]. Here, we observed no significant accumulation of lesions over control at 24 hr after damage induction. The ThyGly and FapyAde levels were consistent with or lower than those in the control; the FapyGua levels reflected a slight, but nonsignificant, increase in lesion content over the control; and there was a slight, but nonsignificant, decrease in 8-oxoGua content. These results are consistent with the loss of oxidatively induced DNA lesions between 4 and 24 hr.

### Cellular GSH Levels

**Figure 6:**
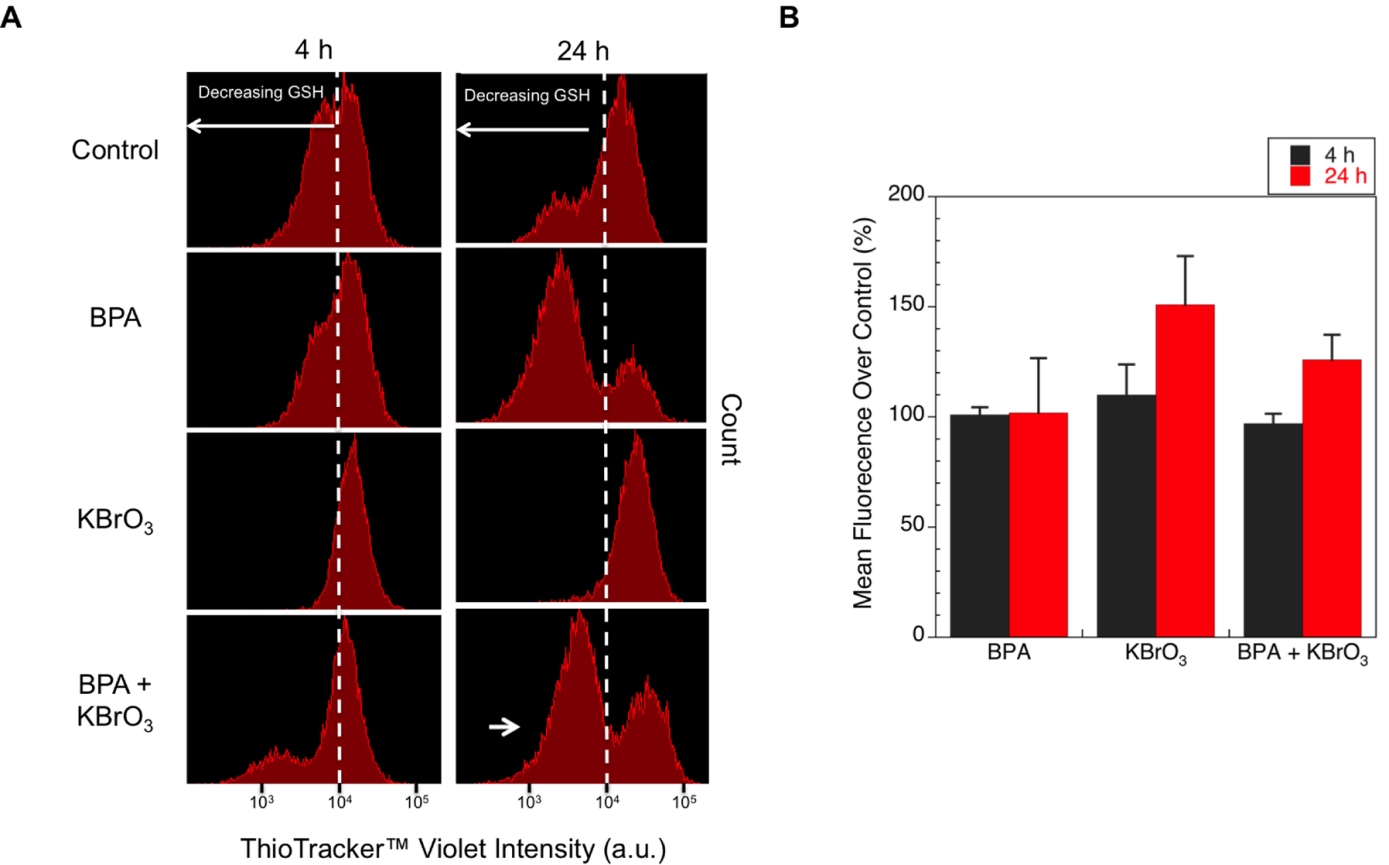
Levels of free GSH after treatment with KBrO_3_, BPA, or co-exposure to both agents at 4 and 24 hr post-damage induction were measured by staining live cells with ThiolTracker™ Violet and sorting by flow cytometry. (*A*) ThiolTracker™ Violet live cells were sorted by intensity, and the measured intensities for a representative experiment at 4 and 24 hr are shown. Dashed lines indicate the center of the intensity peak for the control cells and highlight the relative changes in measured intensity compared with the control cells. The long white arrows indicate the direction of decreasing GSH measured by loss of dye intensity. The short white arrow indicates the subpopulation reflecting lower GSH content. (*B*) Mean intensity values of the ThiolTracker™ Violet staining for each treatment condition 4 hr (black) and 24 hr (red) after damage induction normalized to the control are shown (mean ± SEM of 3 replicates).

In addition to generating oxidatively induced DNA lesions, exposure to oxidative stress can alter the cellular microenvironment and reduce the cellular redox balance. Depletion of intracellular glutathione has been previously observed after BPA exposure (Jain et al. 2011; Kabuto et al. 2003; Wu et al. 2013). To confirm that BPA alters the cellular microenvironment in a time-dependent manner, we measured depletion of intracellular GSH with a fluorescent dye, ThiolTracker™ Violet, which reacts with reduced thiols in live cells. At 4 hr after exposure, the GSH levels in the treated cells were consistent with those in the control, although a small shoulder in the mean intensity profile of the ThiolTracker™ dye appeared in both BPA samples; this indicates that GSH was beginning to be depleted because the fluorescence intensity of the ThiolTracker™ was being reduced in the cell population as a result (Figure 6). At 24 hr after exposure, the GSH levels of cells exposed to BPA were reduced, and a clear second population was observed in the co-exposed cells (Figure 6, indicated by the arrow). Although the flow histograms show the appearance of a second population, a consistent gating could not be found for all treatments to reflect this change in the mean intensity. Therefore, we have presented a representative histogram for the treatments but also report that there were no significant changes observed in the average mean intensity over control for all treatments.

### Intracellular pH

Induction of oxidative stress and depletion of intracellular GSH can also induce changes in intracellular pH. To determine the effects of BPA exposure on intracellular pH, we measured intracellular pH at 4 and 24 hr after treatment (Table 7). At 4 hr post-damage induction, KBrO_3_ alone induced a significant shift in intracellular pH to 6.8. Treatment with BPA alone did not significantly alter the intracellular pH relative to the untreated control. However, BPA co-exposure resulted in a pH of 7.0, which is significantly higher than the pH observed after KBrO_3_ treatment (*p* < 0.05). At 24 hr post-damage induction, the intracellular pH of cells treated with KBrO_3_ alone was similar to the control, whereas the co-exposed cells were significantly acidic compared with the control and the KBrO_3_-only treated cells (*p* < 0.05). The delay in the pH drop may be a result of the depletion of GSH, as shown in Figure 6. Overall, BPA co-exposure appeared to mitigate the pH alterations induced by KBrO_3_-induced oxidative stress at the 4-hr time point.

**Table 7:** Intracellular pH*^a^* in controls and in treated cells (mean ± SD).

Exposure	4 h^*b*^	24 h^*b*^
Control	7.5 ± 0.11	7.5 ± 0.08
BPA	7.5 ± 0.28	7.5 ± 0.11
KBrO_3_	6.8 ± 0.11*	7.8 ± 0.26
BPA + KBrO_3_	7.2 ± 0.08^†^	7.0 ± 0.14*^†^
^***a***^Intracellular pH was quantified by flow cytometry using pHRodo®. ^***b***^Samples treated with 150 μM BPA only were exposed for 5 or 25 hr; those treated with 20 mM KBrO_3_ only were exposed for 1 hr, then medium was replaced for 3 or 23 hr; and co-exposed samples were exposed to 150 μM BPA for 1 hr, then to 150 μM BPA + 20 mM KBrO_3_ for 1 hr, followed by exposure to 150 μM BPA only for 3 or 23 hr. *p < 0.05 compared with untreated controls. ^†^*p* < 0.05 compared with KBrO_3_ alone.		

## Discussion

Numerous reports have indicated that BPA exposure induces global transcriptome and epigenetic changes that can have long-term consequences for cellular regulatory networks and signal transduction pathways (Bromer et al. 2010; Fernandez et al. 2012; Lee et al. 2008; Naciff et al. 2002; Patterson et al. 2015; Ptak et al. 2011; Tabuchi et al. 2006; Weng et al. 2010; Yin et al. 2014). Although dosing conditions and exposure times can be highly variable in the literature, most studies report alterations in DNA response and repair pathways, and a number of studies have shown that BPA exposure induces oxidative stress and oxidatively induced DNA lesions at both environmentally relevant low doses (in the picomolar-nanomolar range) (Koong and Watson 2015; Pfeifer et al. 2015) and at high doses (Babu et al. 2013; Gassman et al. 2015; Jain et al. 2011; Kabuto et al. 2003; Lee et al. 2008; Tiwari et al. 2012; Wu et al. 2013; Yang et al. 2009). Our previous work confirmed these results by revealing that treatment with high doses of BPA and KBrO_3_ alone generated ROS and oxidatively induced DNA lesions [Table 1 and (Gassman et al. 2015)] and by demonstrating an increase in ROS after co-exposure, both through indirect measurement of the generated ROS and through measurement of the significant increase in oxidatively induced lesions (Gassman et al. 2015). However, features of how the cellular microenvironment reacts to BPA-induced oxidative stress and responds to the induced DNA damage have been less well understood. Here, we present evidence that BPA exposure alters the microcellular environment to promote cell survival after the induction of additional oxidative stress by the oxidizing agent KBrO_3_.

Alteration of the chromatin structure through compaction and remodeling has been previously reported after the induction of oxidative stress by hydrogen peroxide (O’Hagan et al. 2011), KBrO_3_ (Amouroux et al. 2010), and after light activation of the KillerRed fluorescent protein (Lan et al. 2014). Further, this compaction has been shown to reduce recruitment of the DNA glycosylase Ogg1, delaying the repair of oxidatively induced DNA lesions (Amouroux et al. 2010), and to promote the suppression of transcription and the accumulation of epigenetic marks silencing genes (O’Hagan et al. 2011). Because high-dose BPA and KBrO_3_ have been shown to induce ROS (Gassman et al. 2015), we evaluated the compaction of chromatin using Hoechst staining. We observed a transient compaction of chromatin 4 hr after exposure to KBrO_3_ and co-exposure to KBrO_3_ and BPA. This compaction may reduce the repair of oxidatively induced DNA lesions, reflected in an increased DNA lesion load consistent with our previous results (Gassman et al. 2015), and may promote the silencing of gene expression, consistent with the down-regulation of DNA repair proteins involved in BER that was observed in the microarray analysis (Table 5).

Coupled with the observed chromatin changes 4 hr post-damage initiation, KBrO_3_/BPA co-exposure also significantly reduced the drop in intracellular pH observed after treatment with KBrO_3_ alone (Table 7). Oxidative stress alters the balance of intracellular redox machinery and can modify cellular membrane ion transport channels (Clerici et al. 1992). Changes in the cellular Na^+^/H^+^ antiporter activity and an increase in intracellular pH have been reported after exposure to estrogen and estradiol (Ediger et al. 1999; Incerpi et al. 2003; Kilić et al. 2009). Here, no increase in intracellular pH was observed after BPA exposure, and to our knowledge, no reports of intracellular pH changes with BPA exposure have been previously reported.

Although depletion of GSH by ROS often results in changes in the cellular Na^+^/H^+^ antiporter activity and is associated with a drop in intracellular pH (Ciriolo et al. 1997; Cutaia and Parks 1994), no significant depletion of GSH was observed 4 hr post-damage induction for any of our treatment conditions (Figure 6). The intracellular pH drop observed after KBrO_3_ treatment was most likely the result of the increase in free K^+^ released upon the formation of the reactive bromate anions. Effects of K^+^ efflux from KCl exposure have been previously described in the literature (Adler and Fraley 1977), although, to our knowledge, our observation of a decrease in intracellular pH following KBrO_3_ exposure is the first. There are numerous possible explanations for how BPA exposure prevents the drop in intracellular pH, from activation of the mitogen-activated protein kinase (MAPK) pathway (Lee et al. 2008) to stimulation of the antiporter system. Further studies are needed to explore the mechanism by which BPA stabilizes against the drop in intracellular pH.

At 24 hr post-damage induction, the suppressive aspects of BPA exposure observed at the 4-hr time point transitioned into cellular microenvironment changes conducive to DNA repair. These changes are consistent with the stimulation of an adaptive response that results in chromatin relaxation in the time period between 4 and 24 hr. At 24 hr, the BPA-treated cells, which showed chromatin compaction compared with the control at 4 hr, were consistent with the control untreated cells; whereas KBrO_3_-treated cells showed compact chromatin at this time point, which may have reflected a progression toward apoptosis (Figure 5). The relaxation of chromatin was also reflected in the microarray results, where up-regulation of DNA repair genes involved in the repair of oxidatively induced DNA damage was observed (Table 4). Finally, the observed reduction in oxidatively induced DNA lesions at 24 hr (Table 6) may indicate the promotion of DNA repair, although further work is required to establish the underlying mechanisms of repair utilized to remove the induced DNA lesions.

Finally, at 24 hr post-damage initiation, a slight, but nonsignificant, reduction in GSH was observed in BPA-exposed cells, and the intracellular pH of the co-exposed cells dropped significantly compared with the control; however, unlike the KBrO_3_-only cells, the pH remained within the range of values observed for the control cells. GSH depletion has previously been observed after exposure to BPA and has been hypothesized to contribute to its prooxidant effects (Babu et al. 2013).

**Figure 7:**
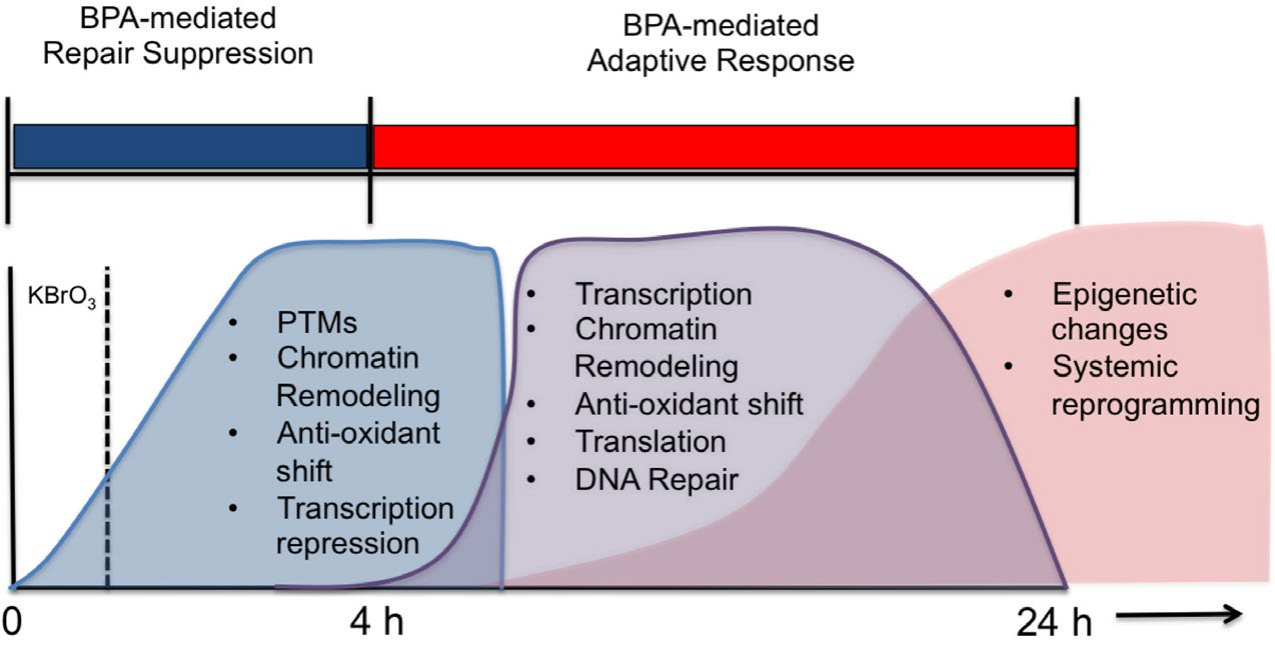
Proposed time-line for the cellular changes observed after BPA exposure based on our findings. We hypothesize that DNA repair is inhibited, at both the recognition and excision levels and at the transcription level up to 4 hr after treatment with BPA (in BPA-only cells) or with KBrO_3_ (in co-exposed cells), and that between 4 and 24 hr, an adaptive response is induced by BPA co-exposure that results in the up-regulation of DNA repair networks, while alterations in the cellular microenvironment are induced through pH changes and antioxidant depletion. Such changes might result in long-term epigenetic changes or reprogramming events. PTM, post-translational modification.

Taken together, these results support a dynamic alteration of the cellular microenvironment in our mouse fibroblast model system that was initiated after co-exposure (Figure 7). Although BPA exposure alone can alter gene expression (Bromer et al. 2010; Fernandez et al. 2012; Lee et al. 2008; Naciff et al. 2002; Patterson et al. 2015; Ptak et al. 2011; Tabuchi et al. 2006; Weng et al. 2010; Yin et al. 2014), deplete GSH (Jain et al. 2011; Kabuto et al. 2003; Wu et al. 2013), and induce oxidative stress in cells (Babu et al. 2013; Xin et al. 2014; Yang et al. 2009), this work demonstrates that high-dose BPA exposure coupled with endogenous or exogenous stresses, such as KBrO_3_-induced oxidative stress, can dramatically alter the microcellular environment and can delay and alter DNA damage response and repair.

These effects are largely uncharacterized in the literature, where the focus has been on examining the endocrine-disrupting functions of BPA or on the characterization of DNA-damaging effects of BPA exposure alone. To that end, it is important to note that our model system utilized normal serum. Our high-dose/short-duration model coupled with normal serum does not directly assay the interactions between BPA and estrogenic chemicals in the serum, nor does it exclude estrogen receptor–mediated effects. However, because the observed cellular effects reported here occur in concert with these other exposures, the fact that mixture effects are complex and poorly understood is further emphasized. Despite the presence of competing estrogenic chemicals, our study nevertheless shows effects of BPA alone that are consistent with those observed in a large number of previous studies (Babu et al. 2013; Jain et al. 2011; Kabuto et al. 2003; Nishimura et al. 2014; Wu et al. 2013; Xin et al. 2014; Yang et al. 2009) Furthermore, the results of our whole genome microarray analysis, when compared with those of other published studies, shows consistency in the gene expression patterns for DNA repair proteins, inflammatory markers, and oxidative stress–related genes that have been observed at much lower doses (Bromer et al. 2010; Fernandez et al. 2012; Lee et al. 2008; Naciff et al. 2002; Patterson et al. 2015; Ptak et al. 2011; Tabuchi et al. 2006; Weng et al. 2010; Yin et al. 2014).

Overall, the cellular responses to KBrO_3_/BPA co-exposure observed in this study are striking, and it is clear from our results that BPA induced oxidative stress and promoted changes to the microcellular environment. These results, although obtained in a cellular model optimized for the study of DNA repair and damage response, have implications for the design of future animal and population-based studies.

We believe that our study highlights the importance of co-exposure effects, especially co-exposure with oxidative stress. The prooxidant activities of BPA and the adaptive response identified here indicate that BPA co-exposure may influence disease development and progression, particularly of inflammatory diseases, which have been linked to BPA exposure (Bindhumol et al. 2003; Chitra et al. 2003; Yang et al. 2009). Future animal model and population-based studies should consider incorporating additional oxidative stress and adaptive response indicators into their study designs to better contextualize the health effects of BPA.

## Conclusions

In our Ku70-deficient MEF model, co-exposure of high-dose BPA with the oxidizing agent KBrO_3_ revealed evidence of changes to the cellular microenvironment that may indicate an adaptive response that promotes cell survival, despite an increase in oxidative stress. We interpret our observations as being consistent with underlying mechanisms whereby BPA-exposed cells undergo an initial period of DNA repair suppression following exogenous damage induced by KBrO_3_, which is followed by an induced adaptive response, unique to the co-exposure condition, that results in whole genome expression changes, chromatin remodeling, depletion of intracellular GSH, and alterations in intracellular pH. However, although we believe that these hypothesized mechanisms are consistent with our observations, they need to be confirmed in other experimental models.

### Appendix 1. Top regulated networks*^a^* 4 hr post-damage induction*^b^*.

KBrO_3_

1. Cell morphology, cellular assembly and organization, cellular function and maintenance
2. Cancer, organismal injury and abnormalities, reproductive system disease
3. Hereditary disorder, neurological disease, lipid metabolism
4. Cell-to-cell signaling and interaction, cellular function and maintenance, hematological system development and function
5. Protein synthesis, RNA post-transcriptional modification, carbohydrate metabolism

BPA

1. Cellular development, cellular growth and proliferation, organ development
2. Cellular movement, immune cell trafficking, connective tissue disorders
3. Cellular development, cellular growth and proliferation, connective tissue development and function
4. Neurological disease, cell-to-cell signaling and interaction, hematological system development and function
5. Dermatological diseases and conditions, organismal injury and abnormalities, lipid metabolism

BPA + KBrO_3_

1. Embryonic development, nervous system development and function, organ development
2. Cellular growth and proliferation, infectious disease, protein synthesis
3. Cell cycle, cellular assembly and organization, reproductive system development and function
4. Developmental disorder, hereditary disorder, metabolic disease
5. Organismal development, tissue morphology, drug metabolism

***^a^***Ranked by IPA by score and number of focus molecules. ***^b^***Samples treated with 150 μM BPA only were exposed for 5 hr; those treated with 20 mM KBrO_3_ only were exposed for 1 hr, then medium was replaced for 3 hr; and co-exposed samples were exposed to 150 μM BPA for 1 hr, then exposed to 150 μM BPA + 20 mM of KBrO_3_ for 1 hr, followed by exposure for 3 hr to 150 μM BPA only.

### Appendix 2. Top regulated networks*^a^* 24 hr after damage induction*^b^.*

KBrO_3_

1. Cardiovascular system development and function, cellular movement, cancer
2. Cell death and survival, dermatological diseases and conditions, developmental disorder
3. Gastrointestinal disease, hepatic system disease, liver cirrhosis
4. Cell death and survival, drug metabolism, endocrine system development and function
5. Cancer, organismal injury and abnormalities, connective tissue disorders

BPA

1. Cellular movement, connective tissue development and function, organ morphology
2. Connective tissue disorders, organismal injury and abnormalities, skeletal and muscular disorders
3. Cardiac dysfunction, cardiovascular disease, organismal injury and abnormalities
4. Organismal development, energy production, molecular transport
5. Cell-to-cell signaling and interaction, cellular movement, hematological system development and function

BPA + KBrO_3_

1. Dermatological diseases and conditions, inflammatory disease, skeletal and muscular disorders
2. Amino acid metabolism, small molecule biochemistry, neurological disease
3. Protein synthesis, cell death and survival, embryonic development
4. Cancer, embryonic development, cellular development
5. Cell cycle, DNA replication, recombination, and repair, cancer

***^a^***Ranked by IPA by score and number of focus molecules. ***^b^***Samples treated with 150 μM BPA only were exposed for 25 hr; those treated with 20 mM KBrO_3_ only were exposed for 1 hr, then medium was replaced for 23 hr; and co-exposed samples were exposed to 150 μM BPA for 1 hr, then exposed to 150 μM BPA + 20 mM of KBrO_3_ for 1 hr, followed by exposure for 23 hr to 150 μM BPA only.

### Appendix 3. Top networks*^a^* regulated by the unique co-exposure genes at 4 and 24 hr after damage induction*^b,c^.*

4 hr

1. Nervous system development and function, organ morphology, organismal development
2. Energy production, nucleic acid metabolism, small molecule biochemistry
3. Developmental disorder, hereditary disorder, metabolic disease
4. RNA post-transcriptional modification, cancer, hematological disease
5. Connective tissue disorders, skeletal and muscular disorders, developmental disorder

24 hr

1. DNA replication, recombination, and repair, hereditary disorder, neurological disease
2. Cancer, gastrointestinal disease, hepatic system disease
3. DNA replication, recombination, and repair, cellular response to therapeutics, cell cycle
4. Gene expression, cancer, hereditary disorder
5. Nucleic acid metabolism, small molecule biochemistry, amino acid metabolism

***^a^***Ranked by IPA by score and number of focus molecules. ***^b^***Samples treated with 150 μM BPA only were exposed for 5 hr; those treated with 20 mM KBrO_3_ only were exposed for 1 hr, then medium was replaced for 3 hr; and co-exposed samples were exposed to 150 μM BPA for 1 hr, then exposed to 150 μM BPA + 20 mM of KBrO_3_ for 1 hr, followed by exposure for 3 hr to 150 μM BPA only. ***^c^***Samples treated with 150 μM BPA only were exposed for 25 hr; those treated with 20 mM KBrO_3_ only were exposed for 1 hr, then medium was replaced for 23 hr; and co-exposed samples were exposed to 150 μM BPA for 1 hr, then exposed to 150 μM BPA + 20 mM of KBrO_3_ for 1 hr, followed by exposure for 3 hr to 150 μM BPA only.
